# Book review: Sally Swartz. 2015. Homeless wanderers: Movement and mental illness in the Cape Colony in the nineteenth century

**DOI:** 10.4102/sajpsychiatry.v22i1.1052

**Published:** 2016-10-26

**Authors:** Rory du Plessis

**Affiliations:** 1 Division of Philosophy and Ethics of Mental Health, University of Pretoria, South Africa

Sally Swartz, one of the foremost South African scholars working in the history of psychiatry, has over two decades published an extensive array of journal articles that have contributed significantly to the debates and discourses of colonial psychiatry. *Homeless Wanderers* is a testament to Swartz’s fluent mastery, expert knowledge and sophisticated scholarship.

The book explores mental illness in the Cape Colony from 1890 to 1910. A central aim of Swartz is to chart ‘a variety of ways in which the insane in the Cape Colony might be studied as a vulnerable population on the move’. Accordingly, the emphasis is on how insane individuals moved between families, jails, asylums, countries, continents and colonies. In this movement to different institutions, sites and contexts, the insane individual came ‘in and out of view’ to family members and authorities: at times, it is the suffering of the individual that is brought into view; at other times, the trauma, suffering, vulnerability and needs for care came out of view and were in many ways discarded, rejected and denied.

Chapter 2 describes colonial psychiatry and its system of care. The construction of a network of lunatic asylums in the Cape Colony aimed to offer humane care to European settler populations. This assembly of asylums also created the possibility of humanitarian care for the indigenous populations. However, this care was negligible in comparison to the treatment offered to the white patients. The chapter chronicles the ways in which colonial psychiatry provided a scientific rationale to buttress the discriminatory and differential degree of care offered to the indigenous patient population.

The complex interactions between lunatic asylums and a number of various other institutions are underpinned in Chapter 3. The Cape Colony was in the most ways faced with a chronic shortage of accommodation for the insane. This meant that asylums worked closely with jails and hospitals to provide temporary accommodation for patients until they could be transferred to an asylum. Moreover, for a large portion of the patients, their route to the asylum was neither direct nor immediate. Some found their way to the asylum after periods in private nursing facilities; domestic homes under the care of family members; and jail following the individual being apprehended for vagrancy, violence or offensive behaviour.

The presence and role of families in the lives of patients in asylum care is the cornerstone of Chapter 4. Using documents from the asylum archive as well as records from local resident magistrates and the district surgeons, Swartz illuminates the degree and nature of the toils and strife that a number of families had with placing a relative into an asylum. Such an exploration aims to evoke the complexity and wide variation of the involvement of families in the lives of their insane relatives.

Chapter 5 provides a panoramic study of the movement of the insane across and into the colony’s borders. It includes accounts of the legislation and discourses designed to police the borders of the colony in order to prevent the immigration of insane persons. Chapter 6 can be regarded as a detailed case study of Jewish immigrants based on the themes established in the previous chapters around ‘travel, families, officials and community responses to insane members’. Chapter 7 not only provides a conclusion but also propounds a number of fresh and compelling insights that will most certainly be underscored in forthcoming scholarship and enquiry.

In this exceptional book, Swartz is commended for her focus on patients as individuals enmeshed in personal, family and community histories rather than constricted to a clinical diagnosis or confined to an institutional history. In this way, we encounter the patient in terms of their individual histories, of the events that preceded or led to their suffering, angst and trauma. Moreover, the encounter with each individual brings into focus the way in which the politics and ideology of the time period impacted on the specific trajectories of each patient.

Swartz’s book can be regarded as an invitation for the psychologist and psychiatrist to reflect on how socio-cultural factors and ideologies influence the practice of psychiatry. The clinician can also draw on the book to raise their understanding of the patients’ viewpoint of distress. For Swartz, this is an opportunity to provide an enriching awareness of how the experience of ‘suffering, turmoil, loneliness, sadness and difficulty accessing containment and care’ in the patient of the 19th century is in some ways connected with the viewpoint of the patients in the present day.

The book will no doubt be an invaluable inspiration for future academic research and should be considered a critical resource for clinicians interested in the history of psychiatry in South Africa.

